# Cystic Fibrosis Mice Develop Spontaneous Chronic *Bordetella* Airway Infections

**DOI:** 10.16966/2470-3176.128

**Published:** 2017-11-02

**Authors:** R Darrah, T Bonfield, JJ LiPuma, P Litman, CA Hodges, F Jacono, M Drumm

**Affiliations:** 1 Frances Payne Bolton School of Nursing, Case Western Reserve University, Cleveland Ohio, USA; 2 Department of Pediatrics, Case Western Reserve University, Cleveland Ohio, USA; 3 Department of Pediatrics and Communicable Diseases, University of Michigan Medical School, Ann Arbor, Michigan, USA; 4 Departments of Radiology, Biomedical Engineering, and Pediatrics, Case Western Reserve University, Cleveland Ohio, USA; 5 Department of Medicine, Case Western Reserve University, and Louis Stokes VA Cleveland Medical Center, USA; 6 Departments of Pediatrics and Genetics Genome Sciences, Case Western Reserve University, Cleveland Ohio, USA

## Abstract

Chronic pulmonary disease and infection is the primary cause of morbidity and mortality in people with cystic fibrosis (CF). Though *Pseudomonas aeruginosa*, is most commonly found in the airways of individuals with CF, there is increasing appreciation for the diversity of the CF microbiome, including other taxa such as *Bordetella*. Here we describe the identification and impact of *Bordetella pseudohinzii* infection in CF mice, which previously have not been thought to develop spontaneous airway infections. We determined that CF mice are more susceptible to the *B. pseudohinzii* infections, and less able to resolve the infection than non-CF mice. Moreover, in both CF and non-CF mice, *B. pseudohinzii* infections lead to markedly reduced respiratory rates and a CF-specific immune response. These results establish the CF mouse model as an important tool for the study of CF-relevant infection and highlight the potential contribution of *Bordetella* to CF clinical pathology.

## Introduction

People with cystic fibrosis (CF) exhibit a complex pulmonary phenotype characterized by airway infection, inflammation, and obstruction. Pulmonary disease, largely a consequence of airway infection with diverse microbiota, accounts for 90% of the mortality in CF.

The most commonly found bacterial species colonizing the lungs of CF patients is *Pseudomonas aeruginosa*, a pathogen not often observed in other patient populations [1]. CF patients infected with *P. aeruginosa* report significantly poorer physical functioning and weight gain, and increased respiratory symptoms and treatment burden, compared to CF patients without *Pseudomonas* infection [2]. As a result of this clinical burden, a great deal of research has focused on the prevalence and treatment of *P. aeruginosa* in CF patients. Recent studies have indicated that the CF airway microbiome is actually much more complicated and diverse, and is typically comprised of many other bacterial species [3,4]. Identification of bacterial species in the airway has historically involved culturing sputum or tracheal swabs and analyzing the bacteria present in the culture. This method is biased, however, based on the culture conditions selected (time, media, temperature), and the competitive ability of the organism to exist in a mixed culture that often contains many different species. Next-generation sequencing technologies provide a less biased method of microbiota detection and identification by eliminating the need for culturing. With this approach, it is clear that microbial communities in the CF airways are diverse, including bacterial species such as *Bordetella* that were previously not typically associated with CF lung infections [5]. *Bordetella* infections can be particularly pathogenic because they are one of a few pathogens known to evade neutrophil extracellular traps, which are a key immune mechanism for respiratory infections [6].

A perceived limitation of CF mouse models has been the lack of spontaneous airway infections by *P. aeruginosa* in these animals. Our previous work describes an increased respiratory rate and alterations in pulmonary mechanics in uninfected CF mice, indicating the presence of a pulmonary phenotype that is not solely a consequence of infection [7,8]. However, in the course of those studies, spontaneous *Bordetella* respiratory tract infections in our CF mouse models were discovered by routine surveillance. Infection was much more common in the CF animals compared to non-CF mice, apparent by the presence of increased neutrophils, lymphocytes, and eosinophils in bronchoalveolar lavage (BAL) fluid and the identity of *Bordetella* was determined by 16S rRNA gene sequencing [5,9]. Initial sequencing results indicated that the bacteria isolated from our mouse colony was *Bordetella hinzii*; however, a more thorough sequence analysis revealed that this was actually a recently described novel species of *Bordetella* referred to as *Bordetella pseudohinzii* [9,10]. *B. pseudohinzii* has since been identified as a confounding species in other mouse models of pulmonary disease [11]. The incidental identification of *B. pseudohinzii* infections in CF and non-CF mice and the effects of *B. pseudohinzii* infection on respiratory rate and infectious response in CF and non-CF mice are presented here.

## Materials and Methods

### Mouse models

A well-described mouse model of CF was utilized for these studies: F508del (*Cftr^tm1kth^*) is a murine version of the most common human CF mutation [12]. These CF mouse models are congenic on the C57BL/6J background, thus, wild-type C57BL/6J were used as controls. Each mouse line is backcrossed to the C57BL/6J background every five generations to minimize possible genetic drift between strains. The Institutional Animal Care and Use Committee of Case Western Reserve University approved the experimental protocols. All mice used in the study were adults (at least 6 weeks old). All mice were allowed unrestricted access to chow (Harlan Teklad 7960; Harlan Teklad Global Diets, Madison, WI) and sterile water with an osmotic laxative, Colyte (Schwarz Pharma, Milwaukee, WI), and were maintained on a 12h light, 12h dark cycle at a mean ambient temperature of 22°C.

### Whole-body plethysmography

Respiratory patterns were recorded in spontaneously breathing mice in a temperature equilibrated whole-body plethysmograph (Sampling rate=200 Hz) following an acclimatization period as described previously [13]. Pressure changes in the chamber were converted to signals representing a ventilatory pattern, passed through a pre-amplifier (Max II, Buxco Electronics), acquired (Power1401, CED, Cambridge, UK) and stored with respiratory acquisition software (Spike 2, CED) for further analysis of breathing-pattern dynamics. The ventilatory pattern was quantified for 60 s epochs. Average values of three epochs are reported. Augmented breaths, sighs, swallows, obvious movements, and gasps were excluded from the analysis, and animals were recorded under comparable conditions.

### Bordetella Infection

Growth curve analysis of *B. pseudohinzii* was done to establish the log-phase of the pathogen to enhance the viability of the inoculum. Mice were inoculated intranasally with 10^6^*B. pseudohinzii* colony-forming units (CFUs), based on the culture and growth pattern of the bacteria. The strain of *B pseudohinzii* used was that originally isolated from our CF mouse colony [5,9]. Mice were followed daily and for up to 2 weeks, and weekly thereafter with weights and clinical score followed by plethysmography and bronchoalveolar lavage (BAL) at varying time points. The BAL fluid was utilized to determine cellular recruitment into the lung and bacterial colonization.

The remaining lungs were also homogenized for bacterial load assessment. The quantification of the bacterial load was done by standard dilution analysis using agar plates, which would also demonstrate any issues with contamination.

### Bronchoalveolar lavage fluid analysis

BAL fluid obtained from mice post-whole body plethysmography was evaluated for total cell count by hemocytometer using four quadrants with the differential evaluated by cytospin and Wright Giemsa Stain. Bacterial cultures were obtained by plating BAL fluid on blood agar plates cultured for 72 hours at 37°C.

### Bordetella ELISA

Mouse serum was diluted 1:50, applied in duplicate to pre-coated *B. hinzii* positive and negative antigen wells (XpressBio), and incubated at 37°C for 30 minutes according to manufacturer’s instructions. Wells were then washed in supplied wash solution (tris buffer with surfactant), blotted dry, and incubated with peroxidase conjugate at 37°C for 45 minutes. Following incubation, wells were washed the last time, blotted dry, and incubated with ABTS substrate at room temperature for 30 minutes. Plates were immediately read for colorimetric change at 405nm on an absorbance plate reader. Negative antigen wells were subtracted as background from positive antigen wells, duplicates averaged, and the results considered positive if abs>0.300 at 405nm, as per manufacturer suggestion.

## Results

*Bordetella* infection was initially detected in the mouse colony by a routine monthly screening of random mice. Once it was detected, we performed a complete broad surveillance using BAL fluid cultures on both CF and non-CF mice. The CF mice in our colony were much more likely to screen positive for infection (66.6% of mice tested) compared to non-CF mice (14.9% of mice tested), despite co-exposure due to housing together. *Bordetella* was detected by BAL fluid culture, indicating respiratory infection at the time of sacrifice, and suggesting that CF mice had either a greater susceptibility for the infection, or a decreased ability to clear it (Table 1). To determine whether the CF mice were more susceptible to infection, we collected longitudinal ELISA data (measuring both IgG and IgM levels) from the CF mouse colony to calculate the percentage of CF and non-CF mice that either were, or ever had been, infected with *Bordetella*. Over a period of approximately 2 years, we screened 180 CF mice, and 137 non-CF mice housed together. 29% of CF mice were positive for *Bordetella* infection, compared to only 2% of non-CF mice tested (Table 2).

**Figure 1:**
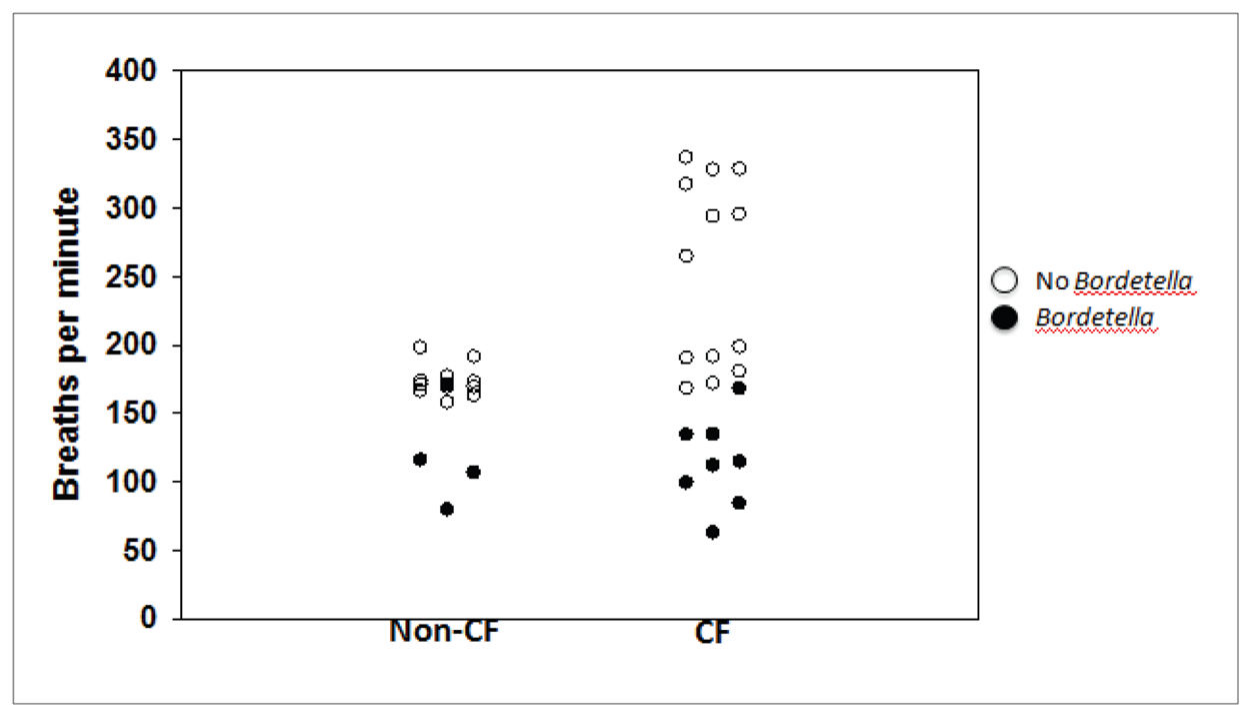
Respiratory rates of non-CF and CF mice are reduced during *Bordetella* infection (112 ± 32 breaths per minute *vs*. 216 ± 64 breaths per minute; p<0.001).

Following the initial outbreak of *Bordetella* infection, we discovered that the CF and non-CF mice that were positive for a current *Bordetella* infection (as determined by BAL culture) also had markedly depressed respiratory rates (112 ± 32 breaths per minute *vs.* 216 ± 64 breaths per minute; p<0.001) (Figure 1). We had measured respiratory rates as part of an ongoing study on a subset of the mice that were initially found to be positive for *Bordetella*. Those results are depicted in Figure 1.

**Figure 2:**
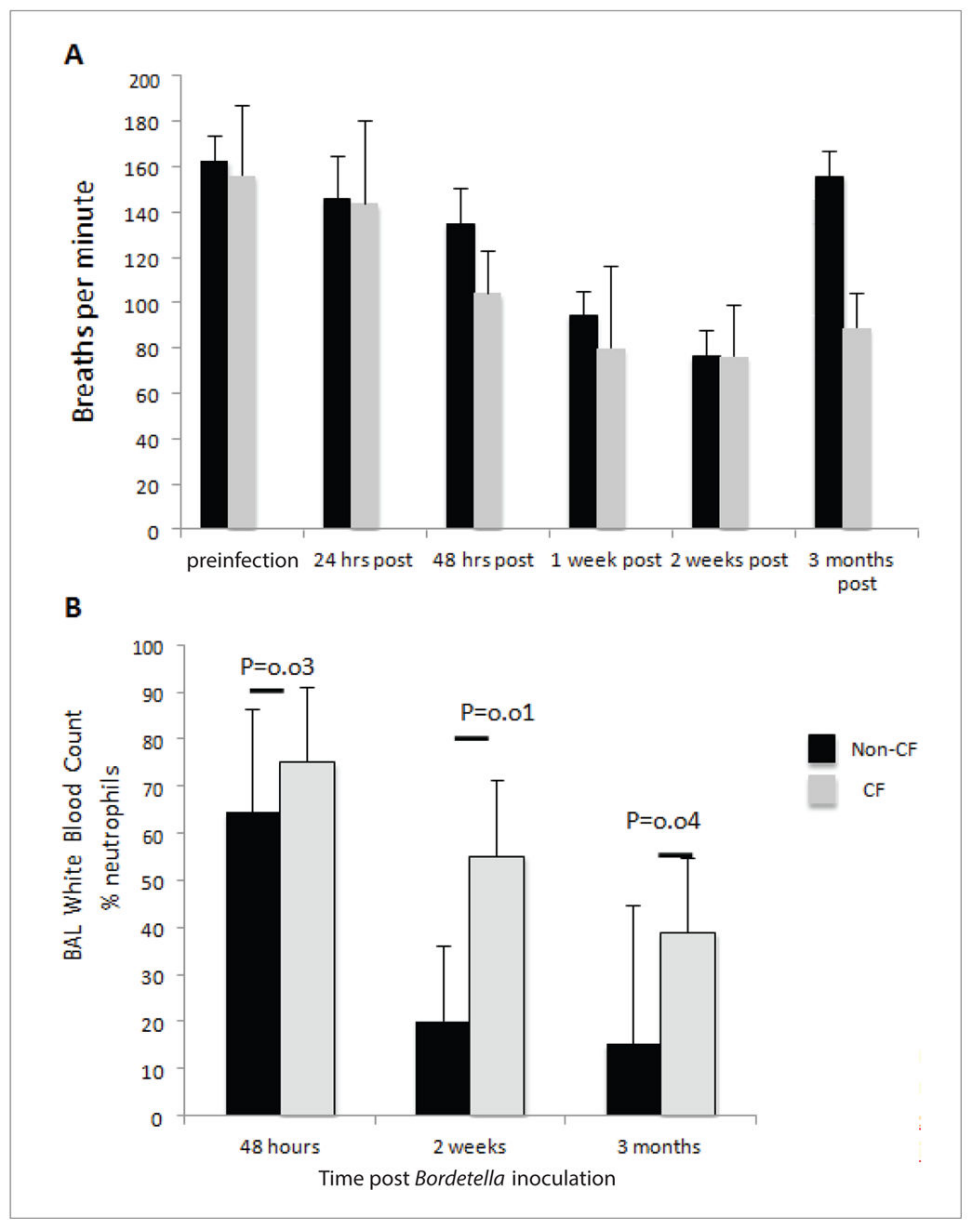
Non-CF (n=12) and CF (n=10) mice both exhibit reduced respiratory rates following *Bordetella* inoculation, which persists 3 months after inoculation in CF mice (A). CF display a significantly increased neutrophilic response to *Bordetella* infection compared to non-CF mice (B).

To evaluate the effects of *Bordetella* infection on respiratory rate and immune response in CF mice, we inoculated uninfected mice intranasally with *B. pseudohinzii* cultures and used plethysmography to monitor the respiratory rates. The respiratory rates of CF mice decreased faster than non-CF mice (48 hours post inoculation *vs*. 1-week post inoculation) and non-CF mice were able to recover from the infection during the 3-month duration of the study, but the CF mice were not (Figure 2A). The CF mice still had markedly reduced respiratory rates compared to non-CF mice 3 months following infection (88 ± 15 breaths per minute *vs*. 155 ± 11 breaths per minute; p<0.01), indicating the effects of the infection on respiratory rate were still present. Following the last measurement of the respiratory rate at 3 months post inoculation, BAL fluid was collected, and 7/9 (78%) CF mice cultured *Bordetella* in their BAL fluid at this time, compared to only 1/11 (9%) of non-CF mice (Table 3).

Finally, we investigated whether there were differences in the immune response of the CF mice compared to non-CF mice. The CF mice had significantly increased percentages of neutrophils in the BAL fluid at 48 hours, 2 weeks, and 3 months post inoculation, indicating a vigorous and prolonged immune response to infection (Figure 2B).

## Discussion and Conclusion

We have described chronic spontaneous lung infection in a CF mouse model due to *Bordetella. Bordetella* infection was detected more often in CF versus non-CF mice, and conferred a dramatically reduced breathing rate in both groups of mice, with the CF mice displaying the reduced respiratory rate at an earlier time point following intentional infection. While the mechanism underlying the reduction in respiratory rate remains to be elucidated, it is important to note that neither the CF nor the non-CF mice exhibited other outward signs of illness or distress. Nevertheless, 78% CF mice still had *Bordetella* cultured from their BAL fluid 3 months following infection, compared to only 9% of non-CF mice. Taken together, these data indicate that CF mice have both an increased susceptibility to *Bordetella* infection, and a decreased ability to clear it.

The CF mice had a higher proportion of neutrophils in BAL fluid at 48 hours, 2 weeks, and 3 months following infection. These data suggest that their inability to clear *Bordetella* pulmonary infection is not a reflection of an inability to mount an immune response following the acquisition of infection. This is consistent with our previous study in which we used a conditional *cftr* allele to eliminate CFTR selectively from myeloid cells and found that those mice have a reduced ability to resolve *Pseudomonas* infection, indicating the absence of CFTR results in impaired immune function [14].

Finally, a majority of the CF mice still had positive *Bordetella* cultures from their BAL fluid 3 months following infection, compared to only one of the non-CF mice. This suggests that the bacteria may be colonized in the CF airway, which is consistent with CF airway disease in humans.

All species of *Bordetella* produce an adenylatecyclase toxin (ACT), dermonecrotic toxin, and tracheal cytotoxin [15]. ACT expression is thought to contribute to respiratory tract colonization [16], dermonecrotic toxin causes tissue damage [17] and tracheal cytotoxin causes ciliary paralysis [18]. The three together are the hallmark of *Bordetella* infections, though there are species-specific toxins, such as pertussis toxin, that can exist in addition to these. The ACT produced by *B. hinzii* differs from other *Bordetella* species (such as *Bordetella pertussis*) in that it has a decreased affinity for calmodulin. As a result, it does not cause the elevated cAMP levels observed in *B. pertussis*-infected patients [15], which is thought to contribute to the differences in virulence between the two species. *B. hinzii* has not been well studied, but it is plausible that though the overall virulence may be reduced compared to other well-studied *Bordetella* species (such as *B. pertussis* and *Bordetella bronchospetica*), infection by *B. hinzii* may have similar, albeit milder effects.

Although *Bordetella* has been identified in the airways of CF patients, it is unclear what role it plays in the pathophysiology of CF lung disease [19–24]. *Bordetella* infections have been estimated to occur in 5–7% of CF patients, though it is thought that the presence of *Bordetella* may be widely underreported due to competition from other species when cultured by standard methods [24]. Direct sequencing of sputum from CF patients revealed the presence of novel species of *Bordetella* [25] in addition to the detection of previously identified strains. Thus, while *Bordetella* has been identified in CF patients [25–27], its contribution to CF lung pathophysiology has not been delineated.

CF patients are typically treated with a myriad of antibiotic courses in an effort to control pulmonary infections. The specific antibiotic treatments are typically selected on the basis of what is cultured from the sputum, with particular attention to the presence of *Pseudomonas* and *Staphylococcus* [28]. *Bordetella* is difficult to culture because it is often competed out at the culture level by other microbial species [24]. The *Bordetella* species isolated from our CF mouse colony is most sensitive to tetracycline and doxycycline. Interestingly, these drugs are typically only used late in the course of CF treatment (on average 20 years after the initial diagnosis), once a patient is diagnosed as having Burkholderia cepacia complex infection [28,29]. Therefore, if a patient with CF has an infection with *Bordetella*, they are unlikely to receive the antibiotic treatment most effective for eradication. *B. hinzii*, the closest relative to *B. pseudohinzii*, has also been found to be sensitive to gentamicin, but not tobramycin [30]. However, gentamicin is not as widely prescribed as tobramycin due to an increased rate of renal toxicity in CF patients. As a result, current clinical interventions for CF are likely to be insufficient in preventing or treating all *Bordetella* infections.

In conclusion, we have described an increased susceptibility to *Bordetella* infection in CF mouse models. This infection causes decreased respiratory rates in both CF and non-CF mice. Interestingly the CF mice, once infected, become chronically so, as they appear unable to clear the infection in spite of their increased neutrophilic response. Whether or not *Bordetella* plays a strong role in the pathogenicity of CF lung infections in humans remains to be determined, but the mouse model offers exciting new avenues for these studies.

## Figures and Tables

**Table 1:** Incidence of spontaneous *Bordetella* infection during initial outbreak determined by BAL culture.

Infection with Bordetella	Non-CF Mice n=67	CF mice n=33
	10/67 (14.9%)	22/33 (66.6%)

**Table 2:** Mice positive for *Bordetella* on ELISA.

Positive ELISA for Bordetella	Non-CF Mice N=137	CF mice N=180
	3/137 (2%)	53/180 (29%)

**Table 3:** Incidence of *Bordetella* infection 3 month post inoculation.

Infection with Bordetella	Non-CF Mice n=11	CF mice n=9
	1/11 (9%)	7/9 (78%)
